# Injecting Jelly Into the Urethra for Sexual Pleasure: A Case Report

**DOI:** 10.7759/cureus.78041

**Published:** 2025-01-27

**Authors:** Mutsuki Morita, Ryo Ichibayashi, Takumi Endo, Hiroyoshi Suzuki

**Affiliations:** 1 Division of Emergency Medicine, Department of Internal Medicine, Toho University Sakura Medical Center, Chiba, JPN; 2 Department of Urology, Toho University Sakura Medical Center, Chiba, JPN

**Keywords:** dangerous sexual practices, liquid foreign bodies, masturbation, penis, scrotum

## Abstract

The insertion of foreign objects into the genitals for sexual pleasure has been reported in a certain number of cases by both men and women. Common foreign objects include household items such as batteries, pens, and thermometers, but unexpected substances such as glue and jelly foods have also been identified. These acts can cause health hazards, and diagnosis is often delayed, especially when patients hide the fact due to shame. This report presents the case of a 54-year-old male who developed bilateral epididymitis after injecting jelly foods into the urethra for sexual pleasure. Initial clinical evaluation was unable to identify the causative substance, even after a medical history, physical examination, and imaging tests, due to the highly radiolucent foreign object. The diagnosis was confirmed by the patient’s detailed medical history and information. The patient’s symptoms improved with antibiotic treatment. Social and psychological factors such as significant stress and economic difficulties may have influenced this behavior. The number of reports of sexual pleasure using highly radiolucent substances such as jelly foods is increasing, and medical professionals need to become more knowledgeable about its health risks. Prompt diagnosis and treatment require detailed interviews and building a relationship of trust. Raising awareness of the health risks associated with such practices is also essential to prevent recurrence.

## Introduction

There are a certain number of cases of self-insertion of foreign objects into the genitals for sexual pleasure, regardless of gender. Some cases have been reported in which people insert foreign objects out of curiosity due to mental illness. The objects inserted range from everyday items, such as batteries, pencils, thermometers, and cucumbers, to items challenging to obtain in the general public, such as bullets and cocaine, and even unexpected items, such as animal bones and glue [1-3]. While these acts bring pleasure, they can also cause various health problems.

In cases of foreign object insertion, a detailed interview is essential. However, many patients try to hide the fact out of shame, and treatment is often delayed. This tendency is particularly noticeable in cases of masturbation for pleasure [1,2,4,5]. In addition to the interview, physical examination and imaging diagnosis are helpful for diagnosis. If the foreign object is radiopaque, it can be confirmed by X-ray or CT scan, and a diagnosis can be made even if information is not obtained from the interview. However, confirmation by imaging tests is complex in the case of liquids or highly radiotransparent substances, and diagnosis may be delayed.

In Japan, the practice of injecting jelly food for energy supply into the bladder through the male genitals to enhance sexual pleasure is widespread in some circles. In this case, the patient developed epididymitis due to the injection of jelly food into the urethra, leading to hospitalization. Although no information on the insertion of a foreign body was obtained during the initial interview, the diagnosis was confirmed by physical examination of the scrotum and repeated interviews. This report describes in detail the masturbation practice using jelly food and the associated health hazards.

## Case presentation

A 54-year-old male had no history of psychiatric disorders and was not taking any medications. He had never smoked and habitually consumed 500 ml of beer daily. He was referred to the emergency department with a one-week history of scrotal pain and swelling. Three days prior to the onset of symptoms, he had experienced fever and cloudy urine but reported no urinary difficulties. There was no recent history of sexual intercourse or use of adult entertainment services.

**Figure 1:**
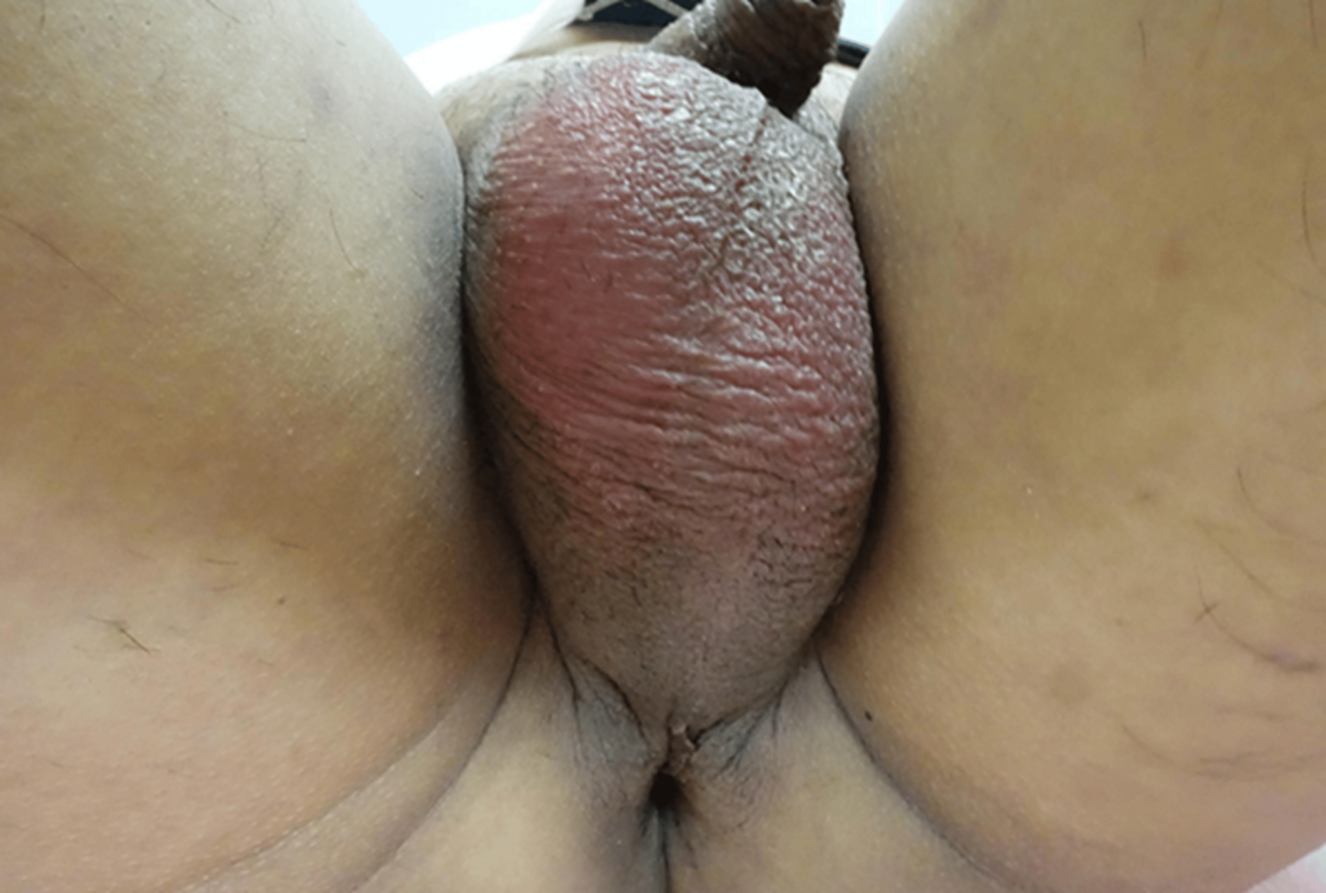
Scrotal findings Redness was observed in the scrotal area. Tenderness was also observed in the exact location.

On arrival, the patient was alert, with a temperature of 36.6°C, blood pressure of 178/112 mmHg, a pulse of 102 beats per minute, and an oxygen saturation of 98% on room air. Physical examination revealed scrotal erythema and tenderness in both testes (Figure 1). Blood and urine tests performed at presentation showed elevated inflammatory markers, mild elevations in biliary enzymes, and bacteriuria (Table 1). No abnormalities were detected in liver function, renal function, or electrolytes. Blood glucose was 114 mg/dl, and hemoglobin A1c was 5.8%.

**Table 1:** Laboratory results upon admission Hb: hemoglobin; Plt: platelet, SG: specific gravity

Test	Result	Unit	Reference range
Blood	-	-	-
CRP	12.95	mg/dL	<0.3
WBC	9,240	/μL	3,300-9,000
Hb	15.7	g/dL	13.5-17.5
Plt	20	10^4^/μL	14-34
Urinalysis	-	-	-
pH	5.5	-	5.0-8.0
SG	1.018	-	1.010-1.025
Glucose	(-)	-	(-)
Protein	(±)	-	(-)
Occult hematuria	(-)	-	(-)
Leukocyte reaction	(2+)	-	(-)
RBC	1-4	/HPF	1-4
WBC	10-19	/HPF	1-4
Squamous epithelium	1-4	/HPF	1-4
Bacteria	(3+)	-	(-)

The patient was suspected of having a testicular infection based on scrotal tenderness and urinalysis findings, prompting the collection of blood and urine cultures. A urologist was consulted, and further interviews revealed that the patient had injected edible jelly into his urethra one week prior. He stated that this practice was undertaken for sexual pleasure through masturbation.

**Figure 2:**
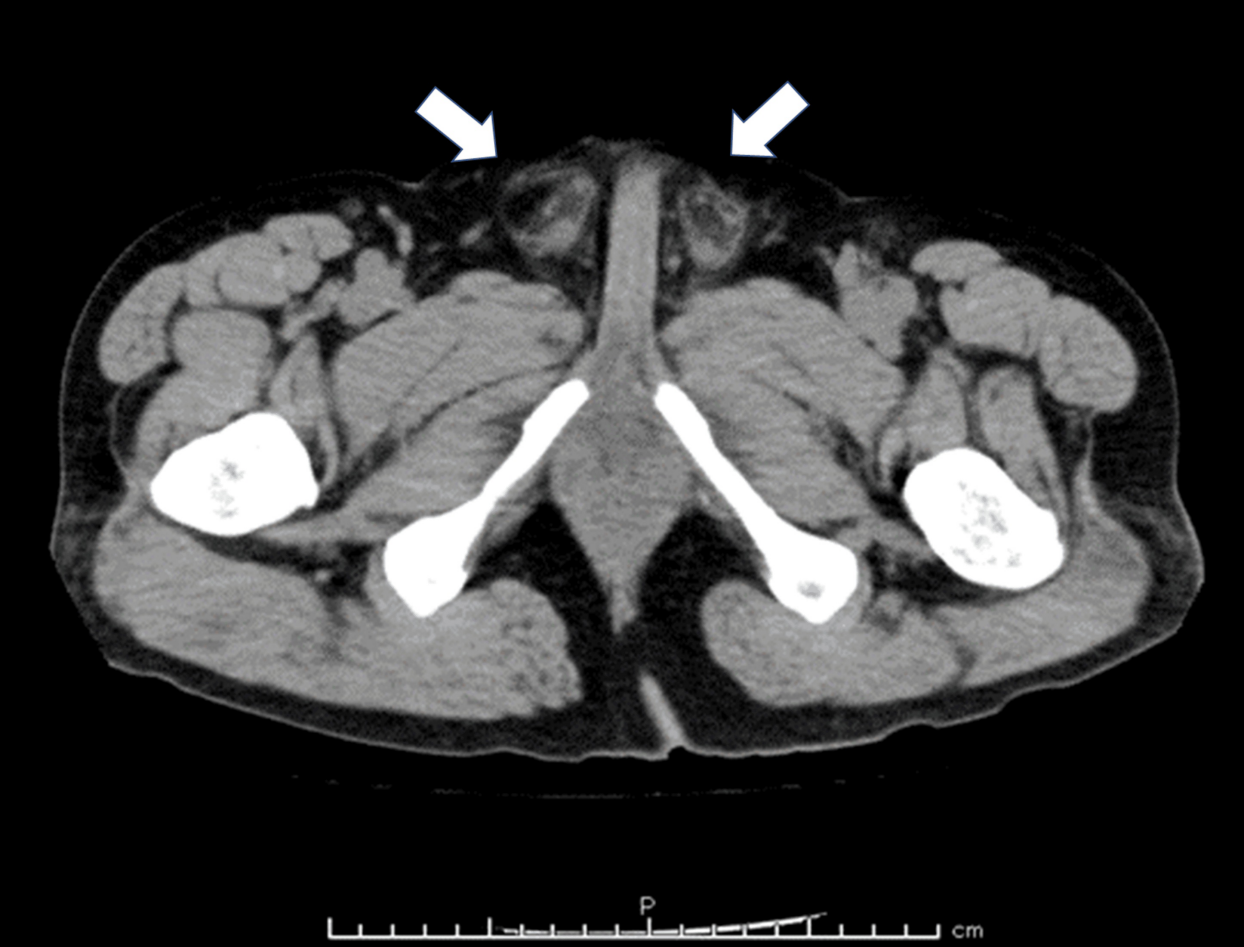
Pelvic CT Increased fat density was observed in both testes (white arrows).

Scrotal ultrasound showed swelling of both epididymides and increased blood flow, with no apparent abnormalities in the prostate or bladder. A plain CT scan of the chest and pelvis revealed bilateral epididymal swelling and inflammatory edema (Figure 2). There was no evidence of prostatic enlargement or inflammation around the prostate, nor were any other apparent causes of inflammation identified. Consequently, a rectal examination was not performed. Based on these findings, a diagnosis of epididymitis due to the injection of a foreign substance was established, and the patient was admitted to the hospital for treatment.

Antibiotic therapy with ceftriaxone (CTRX) 2 g/day was initiated on the first day of hospitalization. However, due to only slight improvement in the inflammatory response, the antibiotics were changed to tazobactam/piperacillin (TAZ/PIPC) 13.5 g/day on the third hospital day. By the fourth hospital day, urine cultures grew *Klebsiella pneumoniae* and *Enterobacter aerogenes*. Blood cultures were negative by the seventh hospital day. Blood tests on the eighth day revealed normalization of WBC counts and improvement in CRP levels. The antibiotic regimen was subsequently adjusted to PIPC 6 g/day on the ninth hospital day.

Despite ongoing antibiotic treatment, scrotal redness, swelling, and pain persisted until the eighth hospital day. Pain was managed with acetaminophen and rofecoxib sodium, leading to improvement by the ninth day, although redness and swelling remained. By the 11th hospital day, mild pain, redness, and swelling persisted in the left scrotum, while the symptoms in the right scrotum improved. On the 13th hospital day, the pain in the left scrotum resolved, although redness and swelling continued.

During hospitalization, an evaluation of the patient’s social background and mental health was conducted. No psychiatric disorders were identified, but his social circumstances were found to be unstable. He had experienced significant stressors, including the death of his mother six months earlier, substantial debt, and unpaid national health insurance premiums. A medical social worker intervened to arrange social support for him after discharge.

On the 14th hospital day, the scrotal swelling and redness had diminished, and inflammatory markers had normalized, allowing for discharge (Table 2). At a follow-up visit six days post-discharge, the swelling and redness had completely resolved, and further outpatient visits were deemed unnecessary.

**Table 2:** Changes in CRP and WBC during hospitalization Changes in CRP and WBC over time are shown. CRP and WBC returned to normal on the 12th day of hospitalization, i.e., the 19th day after the edible jelly injection.

Test	Day 1	Day 3	Day 4	Day 8	Day 12	Day 14	Unit	Reference range
CRP	12.95	11.31	8.41	1.41	0.43	0.22	mg/dL	<0.3
WBC	9,250	12,110	13,510	6,520	6,560	6,560	/μL	3,300-9,000

## Discussion

Sexual activity is one of the basic physiological behaviors of humans, and it has two essential functions: pleasure and reproduction. In particular, sexual pleasure not only brings physiological satisfaction and psychological fulfillment but also plays a vital role in deepening the bond with a partner. It has been reported that sexual pleasure causes the secretion of neurotransmitters such as endorphins and dopamine in the brain, which results in stress reduction, relaxation, and improved self-esteem [6].

Masturbation is widely practiced as a way to obtain sexual pleasure [7]; it is an act of obtaining sexual pleasure through self-stimulation. It is considered a standard behavior among young and middle-aged men because it is expected to have stress reduction and relaxation effects [7]. However, it has also been reported that excessive masturbation and the pursuit of pleasure through inappropriate means can hurt health [1,2,5,8]. In particular, masturbation is often performed in a closed environment, and in some cases, people use foreign objects that others cannot understand. Such use of foreign bodies can cause health problems, and many patients do not seek medical attention until symptoms become severe due to embarrassment [1,4,5,9].

In this case, the patient injected edible jelly into the urethra and masturbated before it reached the bladder, obtaining sexual pleasure. In this act, the patient is said to get pleasure by stimulating the prostate and stimulating the penis up and down when the jelly is injected. It is said to provide greater sexual pleasure than when sperm are excreted. Because jelly is liquid and readily available, it is often mistakenly believed to have little effect on the body. Although reports of urinary tract infections due to the insertion of liquid foreign bodies are rare [1,2,4], this case is an important example that shows the danger.

In conventional cases of foreign body insertion, substances with low radio transparency were often used, which could be easily detected by imaging diagnosis [1,2,4]. On the other hand, when a highly radiotransparent jelly-like food is used, as in this case, it becomes difficult to detect the foreign body by imaging diagnosis, which may result in a delayed diagnosis. Furthermore, it took time for the symptoms of bilateral epididymitis first to become apparent. In this case, epididymitis did not develop immediately after the jelly was injected but created after a certain amount of time, which is also thought to be a factor in the delayed diagnosis. Thus, there are many challenges in the early diagnosis of health damage related to sexual pleasure. To address these challenges, it is essential to disseminate appropriate knowledge and to create an environment for medical consultation that eliminates shame.

In the early stages of treatment, CTRX was administered but was ineffective, and the inflammation improved when it was changed to TAZ/PIPC. This treatment course serves as a guide for the selection of antibiotics in cases of infection caused by inserting a foreign body. Generally, *Neisseria gonorrhoeae *and *Chlamydia trachomatis *are considered to be causative bacteria of epididymitis during sexual activity. Still, in this case, *K. pneumoniae *and *E. aerogenes *were detected by urine culture. The reason why CTRX was ineffective is unclear, but it may have been related to the severe and persistent inflammation caused by the injection of a jelly-like foreign body.

Various factors are said to be related to the act of inserting a foreign body, such as mental illness, intellectual disability, erectile dysfunction, absence of a partner, prison life, and social and economic hardship [2,4,9-11]. In this case, muscular mental stress, such as the death of the mother and financial hardship, may have encouraged the act. Therefore, besides physical treatment, it is essential to provide psychosocial support [4,11]. In this case, an application for welfare assistance was made during hospitalization, and a support system for the future was established.

As a point of reflection, we did not conduct sexually transmitted disease tests such as chlamydia because the patient testified that he had injected the jelly into his urethra for sexual pleasure and claimed to have no history of sexual intercourse. However, it is difficult to completely deny the possibility of sexually transmitted diseases, as patients may not accurately report information about their sexual behavior or may have insufficient awareness of their sexual behavior. In this case, it was challenging to identify the route of infection due to the insertion of a foreign object into the urethra. In such a situation, omitting sexually transmitted disease tests based on the patient’s testimony carries the risk of compromising the accuracy of the diagnosis. Therefore, it is believed that conducting sexually transmitted disease tests as a supplementary measure will contribute to improving the accuracy of determining treatment plans and selecting appropriate antibiotics.

## Conclusions

This case involved bilateral epididymitis caused by the injection of a foreign body (jelly food) into the urethra for sexual pleasure, requiring hospitalization. Shame and stigma often lead patients to hide such behavior, delaying diagnosis and treatment. Radiolucent substances like jelly are challenging to detect with imaging, making thorough interviews essential. The case highlights the role of social and mental stress in such actions and the need for medical teams to address both physical and psychosocial care. A nonjudgmental approach is crucial for building trust and ensuring effective treatment. Public education on the risks of foreign body insertion is vital for prevention.

## References

[1] Jain A, Gupta M, Sadasukhi TC, Dangayach KK (2018). Foreign body (kidney beans) in urinary bladder: an unusual case report. Ann Med Surg (Lond).

[2] Stravodimos KG, Koritsiadis G, Koutalellis G (2009). Electrical wire as a foreign body in a male urethra: a case report. J Med Case Rep.

[3] Mogorovich A, Selli C, Tognarelli A, Manassero F, De Maria M (2018). Intravesical migration of female urethral dilator: a case report of a new urologic emergency in the era of e-commerce. BMC Urol.

[4] Sökmen D, Törer BD, Kargı T, Yavuzsan AH, Şahin S, Tuğcu V (2014). Unusual foreign body in the vesico-urethral; 195 cm liquid pipe. Turk J Urol.

[5] Para SA, Wani SA, Ahmad MS (2021). Management of accidental penile incarceration due to unusual masturbation practices. Turk J Urol.

[6] Esch T, Stefano GB (2011). The neurobiological link between compassion and love. Med Sci Monit.

[7] Fischer N, Træen B (2022). A seemingly paradoxical relationship between masturbation frequency and sexual satisfaction. Arch Sex Behav.

[8] Su J, Wang Y, Feng S, Liao S (2024). Rare vesicourethral foreign body caused by sexual eccentricity - case report. BMC Urol.

[9] Trehan RK, Haroon A, Memon S, Turner D (2007). Successful removal of a telephone cable, a foreign body through the urethra into the bladder: a case report. J Med Case Rep.

[10] Jaiswal AK (1992). An unusual foreign body in the preputial sac. Genitourin Med.

[11] Mahadevappa N, Kochhar G, Vilvapathy KS, Dharwadkar S, Kumar S (2016). Self-inflicted foreign bodies in lower genitourinary tract in males: our experience and review of literature. Urol Ann.

